# The plasminogen receptor, Plg-R_KT_, plays a role in inflammation and fibrinolysis during cutaneous wound healing in mice

**DOI:** 10.1038/s41419-020-03230-1

**Published:** 2020-12-12

**Authors:** Lina Ny, Robert J. Parmer, Yue Shen, Sandra Holmberg, Nagyung Baik, Assar Bäckman, Jessica Broden, Malgorzata Wilczynska, Tor Ny, Lindsey A. Miles

**Affiliations:** 1 Department of Medical Biochemistry and Biophysics, Umeå University, 90187 Umeå, Sweden; 2 Department of Medicine, University of California San Diego, La Jolla, CA USA; 3 Veterans Administration San Diego Healthcare System, San Diego, CA USA; 4 Omnio AB Tvistevägen 48, 90736 Umeå, Sweden; 5 Department of Molecular Medicine, The Scripps Research Institute, La Jolla, CA USA

**Keywords:** Extracellular matrix, Mechanisms of disease

## Abstract

Wound healing is a complex physiologic process that proceeds in overlapping, sequential steps. Plasminogen promotes fibrinolysis and potentiates the inflammatory response during wound healing. We have tested the hypothesis that the novel plasminogen receptor, Plg-R_KT_, regulates key steps in wound healing. Standardized burn wounds were induced in mice and time dependence of wound closure was quantified. Healing in Plg-R_KT_^−/−^ mice was significantly delayed during the proliferation phase. Expression of inflammatory cytokines was dysregulated in Plg-R_KT_^−/−^ wound tissue. Consistent with dysregulated cytokine expression, a significant delay in wound healing during the proliferation phase was observed in mice in which Plg-R_KT_ was specifically deleted in myeloid cells. Following wound closure, the epidermal thickness was less in Plg-R_KT_^−/−^ wound tissue. Paradoxically, deletion of Plg-R_KT_, specifically in keratinocytes, significantly accelerated the rate of healing during the proliferation phase. Mechanistically, only two genes were upregulated in Plg-R_KT_^−/−^ compared with Plg-R_KT_^+/+^ wound tissue, filaggrin, and caspase 14. Both filaggrin and caspase 14 promote epidermal differentiation and decrease proliferation, consistent with more rapid wound closure and decreased epidermal thickness during the remodeling phase. Fibrin clearance was significantly impaired in Plg-R_KT_^−/−^ wound tissue. Genetic reduction of fibrinogen levels to 50% completely abrogated the effect of Plg-R_KT_ deletion on the healing of burn wounds. Remarkably, the effects of Plg-R_KT_ deletion on cytokine expression were modulated by reducing fibrinogen levels. In summary, Plg-R_KT_ is a new regulator participating in different phases of cutaneous burn wound healing, which coordinately plays a role in the interrelated responses of inflammation, keratinocyte migration, and fibrinolysis.

## Introduction

Wound healing is a fundamental and complex physiologic process that proceeds in overlapping, sequential steps^1^. Following burn wounding, vascular permeability increases resulting in the deposition of extravascular fibrin in the wounded area, and in the promotion of the inflammatory phase, which initially consists of infiltration by neutrophils followed by the recruitment of macrophages, the key regulators of the wound healing^2^. Macrophages release pro-inflammatory cytokines to promote additional leukocyte recruitment. Subsequently, the proliferation phase is characterized by keratinocyte proliferation and migration^3^ as well as efferocytosis of apoptotic neutrophils by macrophages and subsequent polarization of macrophages to an anti-inflammatory phenotype, characterized by the secretion of anti-inflammatory cytokines that stimulate the epithelial to mesenchymal transition, activate fibroblasts to proliferate and release collagen and stimulate angiogenesis^4^. In the remodeling phase, granulation tissue is formed^5^.

The plasminogen activation system regulates activation of the circulating zymogen, plasminogen, to the broad spectrum protease, plasmin^6^. Previous studies in plasminogen deficient humans^7–11^ and mice^12–15^ have documented an essential requirement for plasminogen in normal wound healing. Plasmin directly promotes keratinocyte migration^16^ and the role of plasmin was considered to be the promotion of keratinocyte migration by allowing keratinocytes to dissect their way through the fibrin-rich extracellular matrix (ECM) by cleaving components of the ECM^12,17^. However, recent studies from our group have demonstrated a novel key role for plasminogen in wound healing: plasminogen bound to macrophages and neutrophils is transported to the wound area, where the level of plasminogen is increased locally. This leads to the induction of intracellular signaling and cytokine release^18^. Notably, the recruitment of immune cells to cutaneous wounds is not affected by plasminogen deficiency^15,18^ and thus, the role of plasminogen in the initial stages of inflammation is predominantly induction of intracellular signaling. Furthermore, although re-epithelialization eventually occurs in plasminogen deficient mice, the wound area continues to exhibit excessive neutrophil accumulation and collagen deposition after wound closure, providing evidence for an additional requirement for plasminogen in the resolution of inflammation^15^.

Plg-R_KT_ is a transmembrane plasminogen receptor that accounts for the majority of the plasminogen binding capacity of macrophages^19–21^ and promotes plasminogen activation to plasmin by plasminogen activators on cell surfaces^19,22,23^. Plg-R_KT_ is preferentially expressed on proinflammatory monocytes and macrophages^24^. In addition, plasmin-dependent cytokine release from macrophages is promoted by Plg-R_KT_^24,25^.

In the present study, we investigated the role of Plg-R_KT_ in wound healing using a standard burn wound model in mice with global deletion of Plg-R_KT_ as well as in mice with Plg-R_KT_ specifically deleted in either myeloid cells or in keratinocytes. The results of our study suggest that Plg-R_KT_ plays a role in wound healing during the inflammatory, proliferative, and remodeling phases and that Plg-R_KT_ regulates the function of both myeloid cells and keratinocytes during the wound healing process in addition to regulating fibrin clearance during wound healing.

## Materials and methods

### Mice

Mice deficient in Plg-R_KT_ (Plg-R_KT_^−/−^) were generated as described^21^. Plg-R_KT_^+/^^−^ mice were crossed to obtain Plg-R_KT_^−/−^ mice and Plg-R_KT_^+/+^ littermate controls. Mice doubly deficient in Plg-R_KT_ and fibrinogen (Plg-R_KT_^−/−^ /Fgn^−/−^) and littermate controls were produced as described^26^. Plg-R_KT_^*flox/flox*^ mice were generated and were crossed with the B6.129P2-Lyz2tml(cre) Ifo/J strain (Jackson) to obtain the mPlg-R_KT_^−/−^ line as described^25^ and also crossed with Tg(KRT14-cre)randomly 1Amc/J (Jackson 004782) mice to obtain the keratinocyte-deleted (kPlg-R_KT_^−/−^) line. (Deletion of Plg-R_KT_ in keratinocytes isolated from kPlg-R_KT_^−/−^ mice is shown in Supplementary Fig. S1) Male mice age 8–12 weeks were studied. All mice have crossed at least 10 generations into the C57Bl/6 background.

### Burn wound model

Mice were anesthetized with 3% isoflurane (inhaled). Burn wounds (one wound in each mouse) were made with a brass stave and wound healing was quantified as described^18^. Briefly, digital photographs of the wounded skin were taken and analyzed by tracing wound margins and calculating the pixel areas using ImageJ Version 1.41o software (National Institute of Health, Bethesda, USA). The remaining wound area was determined as the percent area of the original wound area. The sample size was chosen based on our previous experience with this model^15,18^. After separating mice by established genotype, mice from each group were assigned randomly to either untreated or burned groups. Blinding: information on genotype was not available to investigators performing burn wounding or to separate investigators who analyzed tissue samples. Burn wounding experiments were performed at least three times.

### Immunohistochemistry and histology

Wounded skin or control unwounded skin was fixed in 4% paraformaldehyde, embedded in paraffin, and sections of 6 µm were cut perpendicular to the skin surface. Immunohistochemistry and histology were performed as in Supplemental Methods.

### Western blotting

Wound tissue was lysed in RIPA buffer with anti-protease and anti-phosphatase cocktail (Thermo Fisher Scientific, Waltham, MA, USA), and western blotting was performed as in Supplemental Methods.

### Quantitative reverse transcription-polymerase chain reaction (RT-PCR)

Skin samples from the wounded area and control unwounded skin (100–200 mg) were cut into 1–2 mm^2^ pieces and kept in RNAlater (Thermo Fischer Scientific) for 3 days at +4 °C and quantitative RT-PCR was carried out as in Supplemental Methods.

### mRNA sequencing

For mRNA sequencing, skin samples were incubated in RNAlater and homogenized in QIAzol (Qiagen, Hilden, Germany) using Precellys CK28R tubes on a Precellys 24 homogenizer. RNA was prepared and characterized as in Supplemental Methods and 5 µg of the pooled RNA was sent to Novogene (Hong Kong) for transcriptome sequencing and data analysis.

### Statistics

A mixed-effects model (REML) was used to analyze repeated measures data due to differences in mouse numbers at different time points. Residual, homoscedasticity, and Q–Q plots were created and inspected to check for heteroscedasticity, normality of residuals, and Gaussian distribution of the dataset to make sure assumptions of repeated measures ANOVA was not violated. Robust ANOVA (rank-based estimation for linear models) which was performed using Rfit package^27^ installed on RStudio (version 1.2.5042^28^) with R 3.6.3^29^, was used to analyze several data sets that involved more than one independent variables for their interaction. Nonparametric comparisons were calculated using GraphPad Prism version 8.0.0 for Windows, GraphPad Software, San Diego, CA, USA. GAPDH normalized PCR data were tested if it was different from the ratio of 1 using the one-sample Wilcoxon test. Time to event data was analyzed in GraphPad Prism, using the Log Rank Mantel–Cox method. All tests were two-tailed.

### Study approval

All animal experiments were approved by the Institutional Animal Care and Use Committee of The Scripps Research Institute and The Regional Ethics Committee of Umeå University.

### Data sharing statement

For original data please contact Tor Ny, tor.ny@umu.se or Lindsey A. Miles, lmiles@scripps.edu. The data from RNA sequencing are available at Figshare: https://figshare.com/account/home#/projects/88871.

## Results

### Plg-R_KT_ deletion decreases healing of burn wounds

**Fig. 1:**
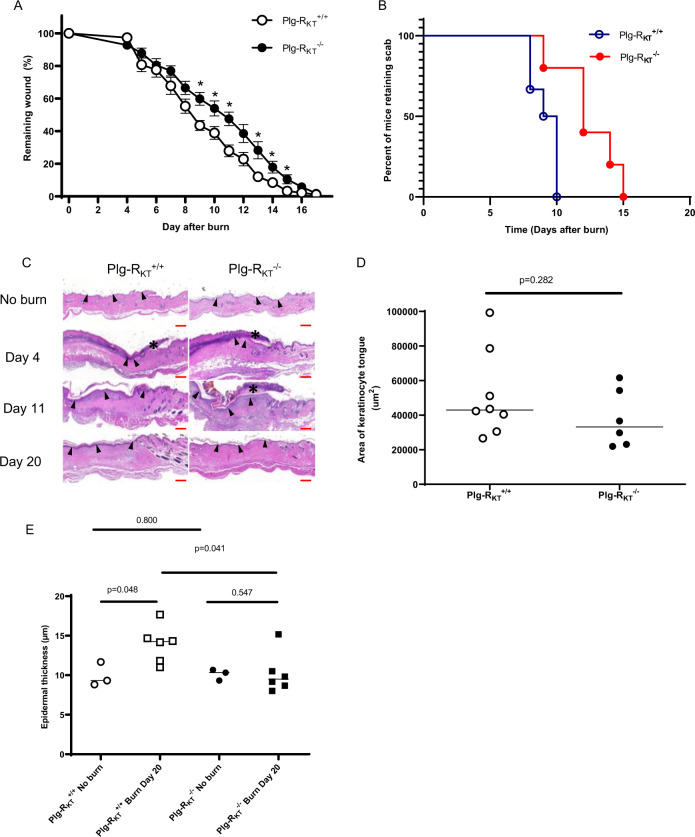
Effect of Plg-R_KT_ deficiency on burn wound healing. **A** Quantification of the remaining wound area at different time points after wounding of male Plg-R_KT_^−/−^ mice (●) and Plg-R_KT_^+/+^ littermates (○) (*n* = 6). The mixed-effects model (REML), showed a significant effect for time (*p* < 0.0001, *f* = 396.6), and for genotype (*p* = 0.040, *f* = 6.00) and a significant time × genotype interaction (*p* < 0.0001, *f* = 4.289). Post hoc testing was done with two-tailed *t* tests at each time point *p < 0.05. **B** Scab loss data are shown for a cohort of Plg-R_KT_^+/+^ mice (*n* = 6) and a cohort of Plg-R_KT_^−/−^ littermates (*n* = 5). *p* = 0.019, *Χ*^2^ = 5.465, d*f* = 1 by the log-rank (Mantel–Cox) test. **C** Representative images of H&E-stained slides of Plg-R_KT_^−/−^ and Plg-R_KT_^+/+^ wound tissue on the day indicated after wounding. The leading edges of the epithelial layer (epithelial tongues) are indicated by open arrows, the epidermal layer is indicated by closed arrows, and the scabs are indicated with asterisks. (On day 4 following wounding, no epidermis was present.) Scale bar = 200 µm. **D** Quantification of data shown in panel C for the area of the tongue at day 4 after wounding. By Mann–Whitney test: Plg-R_KT_^+/+^ (Mdn = 42,984), Plg-R_KT_^−/−^ (Mdn = 33,212), (*U* = 15, *p* = 0.28^2^ *n* = 8 Plg-R_KT_^+/+^ mice and *n* = 6 Plg-R_KT_^−/−^ mice. **E** Quantification of data shown in panel C for epidermal thickness before and at day 20 after wounding. Data analysis by robust ANOVA showed a trend for the effect of genotype (*p* = 0.058, *f* = 4.24), and a trend for the effect of burn treatment (*p* = 0.077, *f* = 3.655) and a burn treatment × genotype interaction (*p* = 0.040, *f* = 5.132). Post hoc testing by Mann–Whitney test showed: Day 0, Plg-R_KT_^+/+^ (Mdn = 9.33), Plg-R_KT_^−/−^ (Mdn = 10.33), (*U* = 3.500, *p* = 0.800); Day 20, Plg-R_KT_^+/+^ (Mdn = 14.25), Plg-R_KT_^−/−^ (Mdn = 9.5), (*U* = 5, *p* = 0.041); Plg-R_KT_^+/+^ Day 0 (Mdn = 9.3), Day 20 (Mdn = 14) (U = 1, *p* = 0.048); Plg-R_KT_^−/−^, Day 0 (Mdn = 10), Day 20 (Mdn = 9^.^5), (U = 6, *p* = 0.547). Images were taken using a Leica DC300F digital camera attached to a Leica DM LB microscope (Leica, Wetzlar, Germany). Epidermal thickness was measured from the photos using Adobe Photoshop. Medians are shown in panels (**A**, **D**, and **E**).

To determine whether deletion of Plg-R_KT_ would affect wound healing rates, full-thickness standardized burn wounds (1 cm in diameter) were induced in Plg-R_KT_^−/−^ male mice and Plg-R_KT_^+/+^ male littermates. Quantification of the wound area at different time points showed that from day 9 after injury, healing in Plg-R_KT_^−/−^ mice was significantly delayed (Fig. 1a). Scab loss was significantly delayed in Plg-R_KT_^−/−^ mice (12th median day of scab loss and 9.5th median day of scab loss for Plg-R_KT_^−/−^ and Plg-R_KT_^+/+^ littermates, respectively (*p* = 0.0194, *n* = 5–6) (Fig. 1b and compare day 11 images in 1C). On day 11, all wounds were covered with an early keratinocyte layer, although some retained a scab (see example in Fig. 1c). The morphologic analysis revealed a decrease in the area of the keratinocyte tongue protruding at wound edges at day 4 (23% less in Plg-R_KT_^−/−^ mice compared with Plg-R_KT_^+/+^ littermates) (Fig. 1d). Interestingly, following wound closure (Day 20), the epidermal thickness was significantly increased in Plg-R_KT_^+/+^ tissue compared with untreated tissue, while epidermal thickness was not increased in Plg-R_KT_^−/−^ tissue following wound closure (Fig. 1c, e). Epidermal thickness was also significantly greater in Plg-R_KT_^+/+^ tissue compared with Plg-R_KT_^−/−^ tissue at day 20, but there was no difference in epidermal thickness between the two genotypes in unburned tissue. These data indicate that Plg-R_KT_^−/−^ mice and Plg-R_KT_^+/+^ mice respond differently to burn treatment with regard to epidermal thickness and may suggest that migration/proliferation of keratinocytes is less in mice deficient in Plg-R_KT_ and that Plg-R_KT_ is required for optimal wound healing.

### Plg-R_KT_ regulates plasminogen accumulation during wound healing

**Fig. 2:**
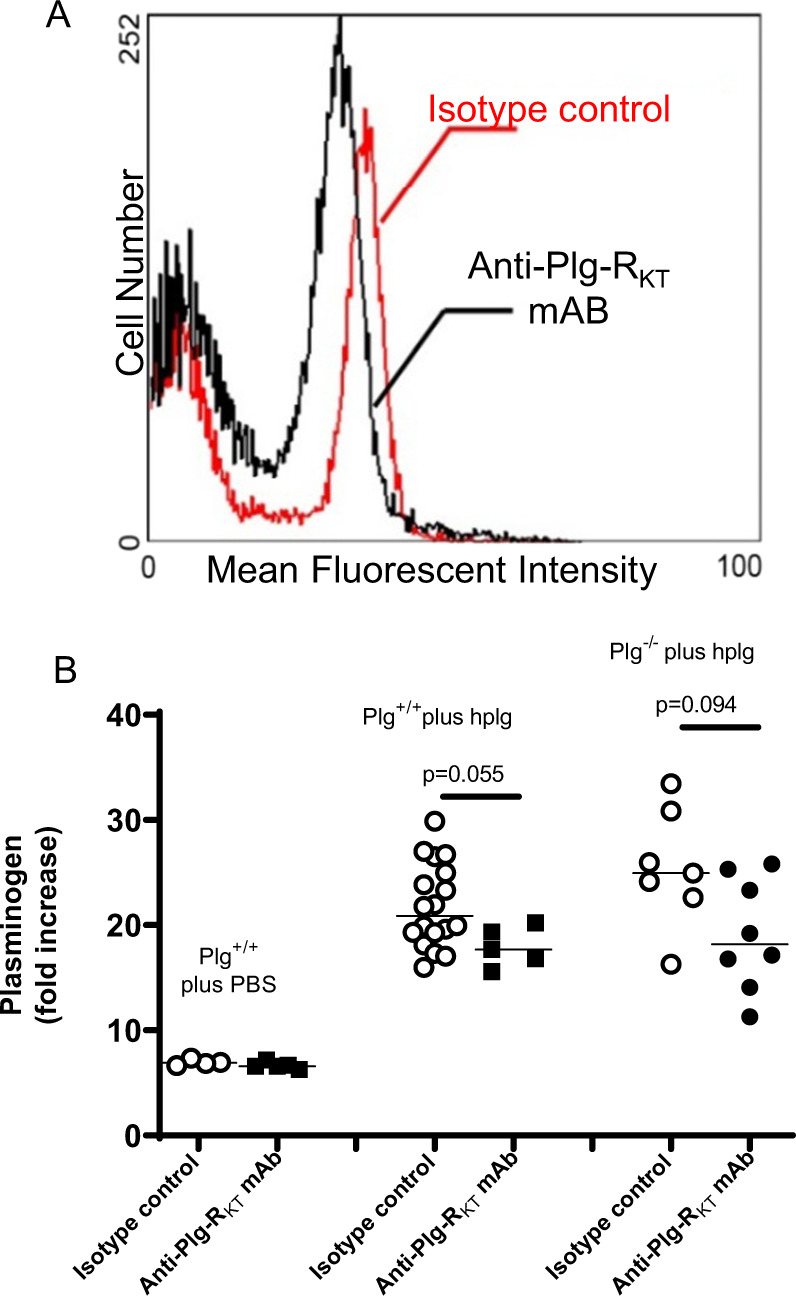
Role of Plg-R_KT_ in plasminogen accumulation in wounds. **A** Effect of anti- Plg-R_KT_ mAB on plasminogen binding to the surfaces of leukocytes. Plg^+/+^ and Plg^−/−^ mice (8–12 weeks of age) were intravenously injected with 100 µl of anti-Plg-R_KT_ mAB7H1 (2.5 mg/ml) or with mouse IgG2A (2.5 mg/ml) as isotype control (n = 3 for each study group). Thirty minutes later, standard burn wounds were introduced and all mice were intravenously injected with 100 µl (2 mg) of Alexa Fluor 488-labeled human plasminogen or PBS (as control). At 24 h after wounding and injection, blood samples were collected. Erythrocytes were lysed immediately with a solution containing 0.15 M NH_4_Cl for 5 min, and the remaining leukocytes were washed and resuspended in 500 μl PBS. FACS analysis was performed using a Cytomics FC500 (Beckman Coulter, Indiana, USA) and leukocytes were defined by forward scatter and side scatter. A clear peak shift of plasminogen binding to leukocytes was observed after anti-Plg-R_KT_ mAB treatment, relative to injection with isotype control. **B** Effect of anti-Plg-R_KT_ mAB on the accumulation of plasminogen in wounds. Both Plg^−/−^ and Plg^+/+^ littermates were injected intravenously with isotype control or anti-Plg-R_KT_ mAB (2.5 mg/ml) 30 min before the burn injury, followed immediately by intravenous injection of human plasminogen (hplg) (2 mg). Wound tissue was collected 24 h after the injury. The plasminogen concentration in wound lysates was determined by specific ELISA. Data analysis by robust ANOVA showed a significant effect of the addition of plasminogen (*p* < 0.001, *f* = 39.649), and a significant effect of antibody treatment (*p* = 0.003, *f* = 9.847), and a trend for antibody treatment × addition of plasminogen interaction (*p* = 0.084, *f* = 2.629). Post hoc testing by Mann–Whitney test showed: Plg-R_KT_^+/+^ plus hplg, isotype control (Mdn = 21), anti-Plg-R_KT_ mAB (Mdn = 18), (*U* = 19, *p* = 0.055); Plg-R_KT_^−/−^ plus hplg, isotype control (Mdn = 24.94), anti-Plg-R_KT_ mAB (Mdn = 8.18), (*U* = 13, *p* = 0.094), *n* = 5–18.

We have previously demonstrated that plasminogen specifically accumulates in burn wounds and that the extent of accumulation directly correlates with circulating plasminogen concentrations^18^. For example, accumulation of plasminogen in burn wounds of heterozygous plasminogen deficient (Plg^+/−^) mice (approximately 50% levels of circulating plasminogen^30^) is reduced by 1.6-fold, compared with wild-type littermates^18^. The transport of plasminogen to wounds is predominantly accomplished by plasminogen binding to plasminogen binding sites on inflammatory cells^18^. It is noteworthy that intravenous injection of 2 mg plasminogen/mouse (to increase circulating plasminogen concentrations by 15.5 µM to a final circulating concentration of 17.5 µM, an ~35-fold increase) results in only a 2.6-fold increase in wound plasminogen accumulation^18^. To detect Plg-R_KT_-dependent effects on plasminogen accumulation in wounds, we used a sensitive antibody inhibition approach. We used our blocking anti-Plg-R_KT_ mAB that decreases recruitment of thioglycolate-elicited macrophages in vivo^23^. First, we verified that anti-Plg-R_KT_ mAB exhibited the ability to block plasminogen binding to leukocytes in vivo. Plg^−/−^ mice were injected with either anti-Plg-R_KT_ mAB or isotype control 30 min before the burn wound was created, followed immediately by injection of Alexa 488-labeled plasminogen. Twenty-four hours later, blood was collected and analyzed by FACS. A clear peak shift of plasminogen binding to leukocytes was observed after anti-Plg-R_KT_ mAB treatment, relative to injection with isotype control, indicating that anti-Plg-R_KT_ mAB blocked plasminogen binding to leukocytes in vivo (Fig. 2a). When plasminogen accumulation in the burn wounds was analyzed following injection of plasminogen into Plg^+/+^ and Plg^−/−^ mice, a significant effect of treatment with anti-Plg-R_KT_ mAB was shown by analysis with robust ANOVA (*p* = 0.003, *f* = 9.847) (resulting in an 18% and a 25% decrease for Plg^+/+^ and Plg^−/−^ mice, respectively) (Fig. 2b). Significant effects of treatment with anti-Plg-R_KT_ mAB on plasminogen accumulation (Fig. 2b) in wound tissue of mice that received PBS were not detectable. Thus, under these conditions, anti-Plg-R_KT_ mAB blocked the ability of cells to transport human plasminogen to the wounds, but the ability of anti-Plg-R_KT_ mAB to dissociate pre-bound mouse plasminogen was not detectable. Our data suggest that Plg-R_KT_ allows cells to transport plasminogen to wound sites.

### Plg-R_KT_ expression increases during wound healing

**Fig. 3:**
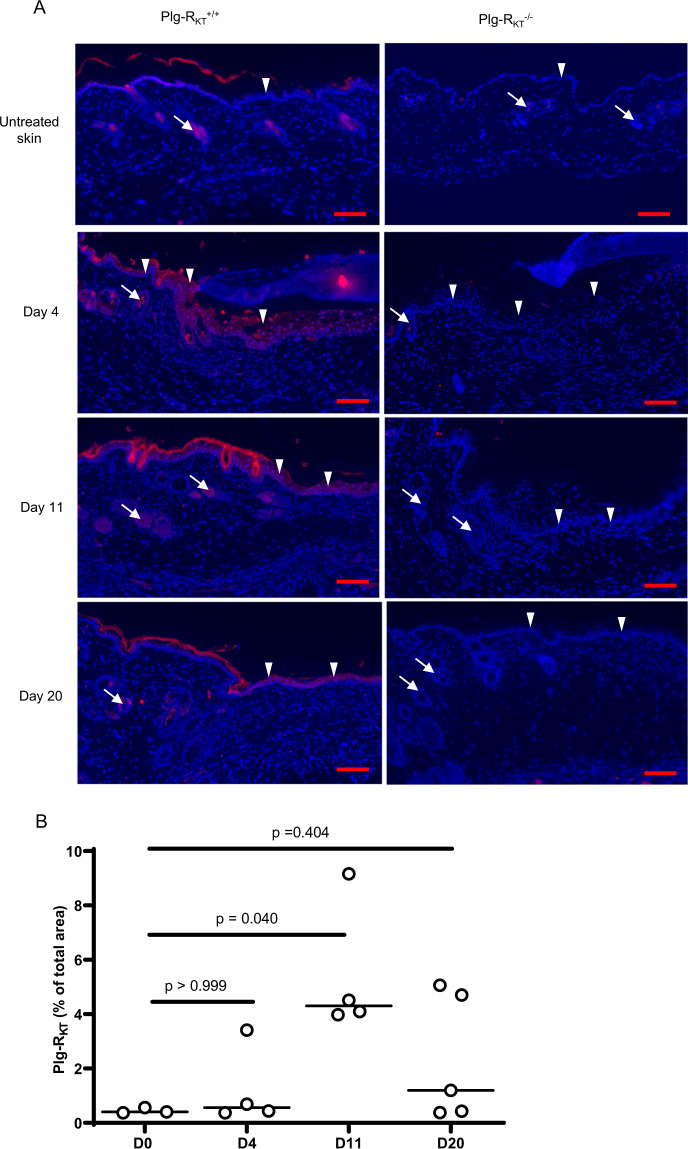
Expression of Plg-R_KT_ in wound tissue. **A** Paraffin sections of wound tissue from Plg-R_KT_^−/−^ and Plg-R_KT_^+/+^ littermates were immunostained with anti-Plg-R_KT_ mAB (red) and DAPI (blue). Scale bar = 200 µm. White arrows indicate hair follicles, white arrowheads show keratinocytes. **B** Quantitation of the Plg-R_KT_ staining in Plg-R_KT_^+/+^ (shown as % of total wound area). By Krustal–Wallis test, *p* = 0.054, Krustal–Wallis statistic = 7.1 followed by Dunns Multiple Comparisons, Day 0 vs. Day 4, *p* ≥ 0.999, Day 0 vs. Day 11 *p* = 0.040 and Day 0 vs. Day 20, *p* = 0.404, (*n* = 4). Medians are displayed. Images were captured with a Zeiss Axio Imager Z1 (Zeiss, Oberkochen, Germany) or Nikon A1R Eclipse Ti-E inverted microscope (Nikon Instruments, Amsterdam, Netherlands). Quantification of fluorescent areas was performed using Image J software.

We examined the expression and localization of Plg-R_KT_ in wounded skin. Expression of Plg-R_KT_ increased during wound healing and was maximal at day 11 and decreased by day 20 (Fig. 3a, left panels, and quantified in Fig. 3b). Plg-R_KT_ was localized in hair follicles in unwounded skin (white arrows) and was prominently expressed in both hair follicles and keratinocytes (white arrowheads) on days 4, 11, and 20 following wounding. Plg-R_KT_ expression was also confirmed in isolated primary keratinocytes (Supplementary Fig. S1). Plg-R_KT_ expression was not detected in wound tissue in Plg-R_KT_^−/−^ littermates, as negative controls for the immunostaining (Fig. 3, right panels).

### Specific deletion of Plg-R_KT_ in myeloid cells decreases the rate of wound healing during the transition from the inflammatory phase to the proliferation phase

**Fig. 4:**
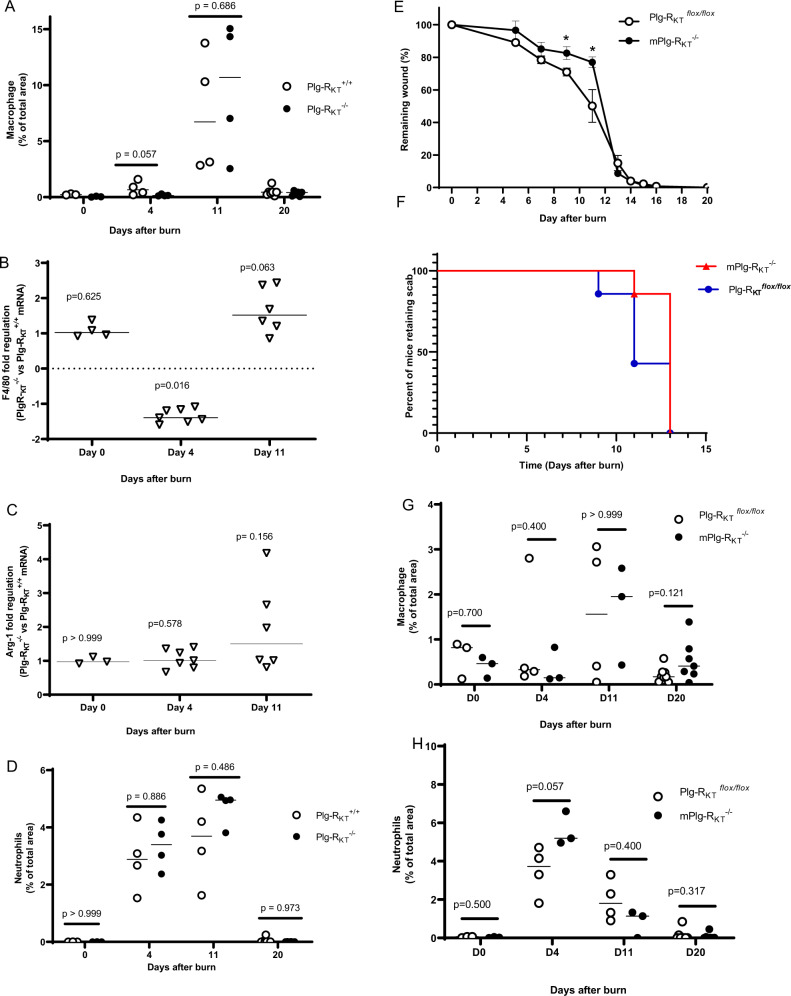
Role of macrophage/neutrophil Plg-R_KT_ in burn wound healing. **A** Quantification of CD68 staining of the burn wound tissue from Plg-R_KT_^−/−^ and Plg-R_KT_^+/+^ littermates for macrophages, (*n* ≥ 3). Robust ANOVA showed a significant effect for time (*p* < 0.0001, *f* = 24.180), but not for genotype (*p* = 0.759, *f* = 0.096) or for time × genotype interaction (*p* = 0.782, *f* = 0.361). Post hoc testing by Mann–Whitney test showed: Day 0, Plg-R_KT_^+/+^, (Mdn = 0.21), Plg-R_KT_^−/−^ (Mdn = 0.033), (*U* = 0, *p* = 0.100); Day 4, Plg-R_KT_^+/+^ (Mdn = 0.659), Plg-R_KT_^−/−^ (Mdn = 0.140), (*U* = 1, *p* = 0.057); Day 11, Plg-R_KT_^+/+^ (Mdn = 6.73), Plg-R_KT_^−/−^ (Mdn = 10.7), (*U* = 6, *p* = 0.686); Day 20, Plg-R_KT_^+/+^ (Mdn = 0.445), Plg-R_KT_^−/−^ (Mdn = 0.418), (*U* = 15, *p* = 0.445). **B** qPCR analysis of burn wound tissue from Plg-R_KT_^−/−^ and Plg-R_KT_^+/+^ littermates for expression of F4/80. By One-Sample Wilcoxon test: Day 0, (Mdn = 1.022), (*W* = 4.000, *p* = 0.625); Day 4, (Mdn = −1.395), (*W* = −28.00, *p* = 0.016); Day 11, (Mdn = 1.518), (*W* = 19.00, *p* = 0.063), *n* = 4–7. **C** qPCR analysis of burn wound tissue from Plg-R_KT_^−/−^ and Plg-R_KT_^+/+^ littermates for expression of arginase 1. By the One-Sample Wilcoxon Test: Day 0, (Mdn = 0.973), (*W* = 0.000, *p* > 0.999); Day 4, (Mdn = −1.009), (*W* = 8.000, *p* = 0.578); Day 11, (Mdn = 1.504), (*W* = 15.000, *p* = 0.156). **D** Quantification of Ly6B.2 staining of the burn wound tissue from Plg-R_KT_^−/−^ and Plg-R_KT_^+/+^ litter mates for neutrophils (n ≥ 3). Robust ANOVA showed a significant effect for time (*p* < 0.0001, *f* = 79.548), and for genotype (*p* = 0.006, *f* = 8.765) and a significant time × genotype interaction (*p* = 0.035, *f* = 3.318). Post hoc testing by Mann–Whitney Test: Day 0, Plg-R_KT_^+/+^ (Mdn = 0.00045), Plg-R_KT_^−/−^ (Mdn = 0.002), (*U* = 4, *p* > 0.999); Day 4, Plg-R_KT_^+/+^ (Mdn = 2.9), Plg-R_KT_^−/−^ (Mdn = 3.4), (*U* = 7, *p* = 0.886); Day 11, Plg-R_KT_^+/+^ (Mdn = 3.7), Plg-R_KT_^−/−^ (Mdn = 5.0), (*U* = 5, *p* = 0^.^486); Day 20, Plg-R_KT_^+/+^ (Mdn = 0.006), Plg-R_KT_^−/−^ (Mdn = 0.009), (*U* = 21, *p* = 0.973). **E** Quantification of the remaining wound area at different time points after wounding of male mPlg-R_KT_^−/−^ mice and Plg-R_KT_^*flox*/*flox*^ mice (*n* = 7), The mixed-effects model (REML), showed a significant effect for time (*p* < 0.0001, *f* = 491.8), and for genotype (*p* = 0.066, *f* = 4.028) and a significant genotype × time interaction (*p* < 0.0001), *f* = 5.213). Post hoc testing by two-tailed *t* tests *p < 0.05. Means ± S.E.M. is shown. **F** Scab loss data are shown for a cohort of Plg-R_KT_^*flox*/*flox*^ mice and a cohort of mPlg-R_KT_^−/−^ mice (*n* = 7). *p* = 0.0974, *Χ*^2^ = 2.747, d*f* = 1 by the log-rank (Mantel–Cox) test. **G** Quantification of CD68 staining of the burn wound tissue from mPlg-R_KT_^−/−^ and Plg-R_KT_^*flox/flox*^ littermates for macrophages (*n* ≥ 3). Data analysis by robust ANOVA showed a trend for effect for time (*p* = 0.053, *f* = 2.912), but no effect for genotype (*p* = 0.829, 0.047) or for a genotype × time interaction (*p* = 0.589, *f* = 0.651). Post hoc testing by Mann Whitney: Day 0, Plg-R_KT_^*flox/flox*^ (Mdn = 0.819), mPlg-R_KT_^−/−^ (Mdn = 0.461), (*U* = 3, *p* = 0.700); Day 4, Plg-R_KT_^*flox/flox*^ (Mdn = 0.329), mPlg-R_KT_^−/−^ (Mdn = 0.147), (*U* = 3, *p* = 0.400); Day 11, Plg_-_R_KT_^*flox/flox*^ (Mdn = 1.562), mPlg-R_KT_^−/−^ (Mdn = 1.950), (*U* = 6, *p* > 0.999); Day 20, Plg_-_R_KT_^*flox/flox*^ (Mdn = 0.173), mPlg-R_KT_^−/−^ (Mdn = 0.408), (*U* = 14, *p* = 0.121). **H** Quantification of Ly6B.2 staining of burn wound tissue from mPlg-R_KT_^−/−^ and Plg-R_KT_^*flox/flox*^ littermates for neutrophils (*n* ≥ 3). Data analysis by robust ANOVA showed a significant effect for time (*p* < 0.0001, f = 62.655), but no effect for genotype (p = 0.219, f = 1.582) and a significant genotype × time interaction (*p* = 0.001, *f* = 7.075). Post hoc testing by Mann–Whitney test: Day 0, Plg-R_KT_^*flox/flox*^ (Mdn = 0.059), mPlg-R_KT_^−/−^ (Mdn = 0.019), (*U* = 2.5, *p* = 0.500); Day 4, Plg-R_KT_^*flox/flox*^ (Mdn = 3_._7), mPlg-R_KT_^−/−^ (Mdn = 5.2), (*U* = 0, *p* = 0.057); Day 11, Plg-R_KT_^*flox/flox*^, (Mdn = 1.8), mPlg-R_KT_^−/−^ (Mdn = 1.1), (*U* = 3, *p* = 0.400); Day 20, Plg-R_KT_^*flox/flox*^ (Mdn = 0.0045_)_, mPlg-R_KT_^−/−^ (Mdn = 0.0090), (*U* = 19, *p* = 0.317). Images were captured with a Zeiss Axio Imager Z1 (Zeiss, Oberkochen, Germany) or Nikon A1R Eclipse Ti-E inverted microscope (Nikon Instruments, Amsterdam, Netherlands). Quantification of fluorescent areas was performed using Image J software. For panels **A**–**D**, **G**, and **H** medians and *p* values from post hoc testing by Mann–Whitney test are displayed.

We have shown previously that the bulk of plasminogen transport to wound sites is accomplished by inflammatory cells^18^. Therefore, we used immunohistochemistry and quantified the percentage of macrophages and neutrophils in the wounds at Days 4, 11, and 20 after burning. Macrophage recruitment to the wound sites was time-dependent and peaked at day 11 following wounding, but the genotypes did not respond differently to burn treatment with regard to time (Fig. 4a). There was also no genotype effect on the expression of the macrophage markers, F4/80 and arginase 1, as determined by qPCR (i.e., fold difference in expression was not greater than 1.6 at each time point tested (Fig. 4b, c, respectively), consistent with results of the immunohistochemical analyses. Neutrophils were most abundant in the wounds at days 4 and 11 following wounding, but there also was no significant genotype effect on the total number of neutrophils present at each time point (Fig. 4d).

In view of the effect of anti-Plg-R_KT_ mAB in reducing plasminogen levels in wounds of both Plg^+/+^ and Plg^−/−^ mice (Fig. 2b), we directly assessed the potential role of macrophage/neutrophil Plg-R_KT_ in wound healing. Burn wounds were induced in mPlg-R_KT_^−/−^ mice, in which Plg-R_KT_ was specifically deleted in myeloid cells^25^, and Plg-R_KT_^flox/flox^^25^ control mice. Quantification of the wound area at different time points showed that the genotypes responded differently to burn wounding with respect to time (*p* < 0.0001) with a prominent delay in wound healing at days 10 and 12 (Fig. 4e) during the proliferation phase, when the maximal number of macrophages were present in the wounds of mice with global deletion of Plg-R_KT_ (Fig. 4a), during the transition from the inflammatory to proliferation phases of wound healing. There was also a trend for delayed scab loss in mPlg-R_KT_^−/−^ wounds (13th median day of scab loss and 11th median day of scab loss for mPlg-R_KT_^−/−^ and Plg-R_KT_^flox/flox^ mice, respectively, *p* = 0.097, *n* = 7) (Fig. 4f). Macrophage recruitment to mPlg-R_KT_^−/−^ wound sites peaked at day 11 following wounding, but there was no significant effect of myeloid-specific deletion of Plg-R_KT_ on macrophage accumulation at this time point, nor at earlier time points examined (Fig. 4g). Neutrophils were most abundant in the wounds on day 4 following wounding, but there also was no significant effect of cell-specific deletion of Plg-R_KT_ on the total number of neutrophils present at each time point (Fig. 4h).

### Specific deletion of Plg-R_KT_ in keratinocytes increases the rate of wound healing and results in a thinner epidermis

**Fig. 5:**
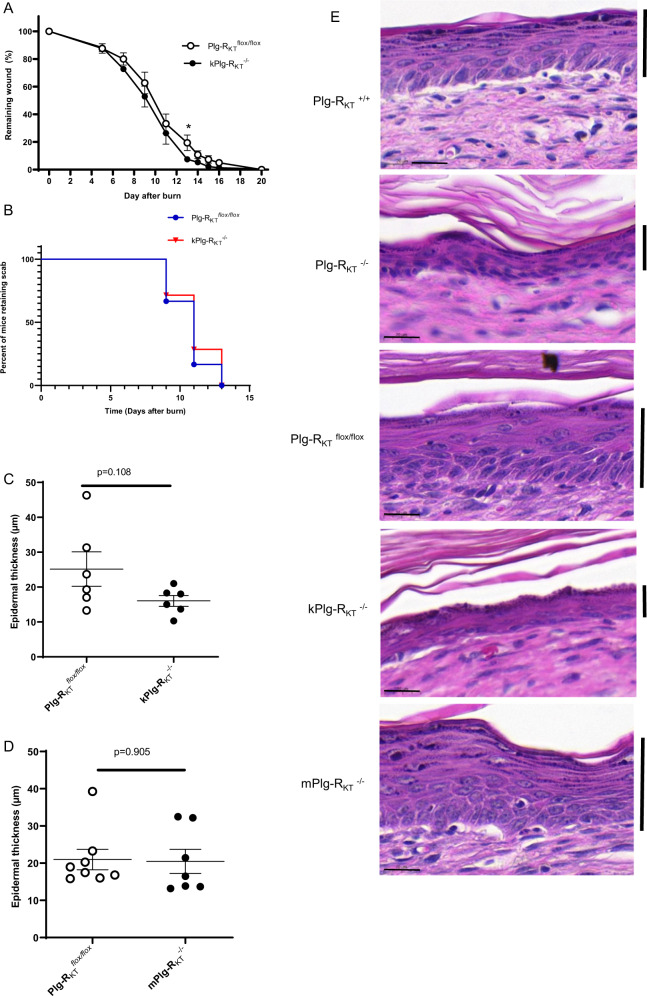
Role of keratinocyte Plg-R_KT_ in burn wound healing. **A** Quantification of the remaining wound area at different time points after wounding of male kPlg-R_KT_^−/−^ mice and Plg-R_KT_^*flox*/*flox*^ mice (*n* ≥ 7). The mixed-effects model (REML), showed a significant effect for time (*p* < 0.0001, *f* = 211.9), and a trend for genotype (*p* = 0.103, *f* = 3.158) but no significant genotype × time interaction (*p* = 0.745), *f* = 0.658). Post hoc testing by two-tailed *t* test *p = 049. **B** Scab loss data are shown for cohorts of Plg-R_KT_^*flox*/*flox*^ and kPlg-R_KT_^−/−^ mice) (*n* = 7). *p* = 0.669, *Χ*^2^ = 0.182, d*f* = 1 by the log-rank (Mantel–Cox) test. **C** Quantification of epidermal thickness at day 20 after wounding of kPlg-R_KT_^−/−^ and Plg-R_KT_^*flox*/*flox*^ mice. **D** Quantification of epidermal thickness at day 20 after wounding of mPlg-R_KT_^−/−^ and Plg-R_KT_^*flox*/*flox*^ mice. Means ± S.E.M. determined by two-tailed *t* test is shown in (**A**, **C**, **D**). **E** Representative images of H&E stained sections taken from the middle part of the healed area at day 20 after wounding from mice with different genotypes. Scale bar = 20 µm. The keratinocyte layer is marked with a vertical bar. Images were taken using a Leica DC300F digital camera attached to a Leica DM LB microscope (Leica, Wetzlar, Germany). Epidermal thickness was measured from the photos using Adobe Photoshop.

In view of the expression of Plg-R_KT_ in keratinocytes (Fig. 3 and Supplemental Fig. 1) and effects of global Plg-R_KT_ deletion on epidermal thickness (Fig. 1e), we assessed the potential role of keratinocyte Plg-R_KT_ in wound healing in mice with Plg-R_KT_ specifically deleted in keratinocytes (kPlg-R_KT_^−/−^). Burn wounds were induced in kPlg-R_KT_^−/−^ mice and Plg-R_KT_^flox/flox^ control mice. Quantification of the wound area at different time points did not reveal a significant difference between the genotypes in response to burn to wound with respect to time. However, healing in kPlg-R_KT_^−/−^ mice was significantly accelerated at day 13 during the proliferation phase (Fig. 5a). There was no effect of specific deletion of Plg-R_KT_ in keratinocytes on scab loss, consistent with an effect of specific keratinocyte deletion of Plg-R_KT_ only during the proliferation phase (Fig. 5b). There was a trend for decreased epidermal thickness in wounds of kPlg-R_KT_^−/−^ mice compared with Plg-R_KT_^flox/flox^ control mice (Fig. 5c). In contrast, there was no detectable effect of specific myeloid deletion of Plg-R_KT_ on epidermal thickness compared to the Plg-R_KT_^*flox/flox*^ control (Fig. 5d). Thus, keratinocyte Plg-R_KT_ appears to regulate wound healing during the proliferation phase.

### Effects of Plg-R_KT_ deletion on gene expression during wound healing

**Fig. 6:**
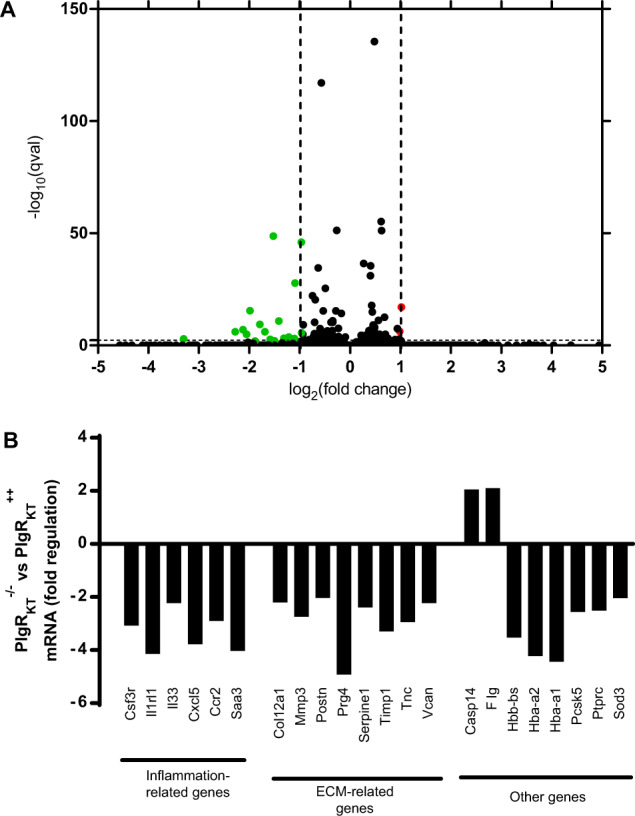
Gene expression in Plg-R_KT_^−/−^ and Plg-R_KT_^+/+^ wound tissue. Gene expression was studied using mRNA sequencing in samples taken at day 11 (*n* = 4). All the genes had corrected *p* value ≤ 0.05. **A** Volcano plots with genes upregulated > 2.0-fold in Plg-R_KT_^−/−^ tissue shown in red, genes downregulated <2.0-fold Plg-R_KT_^−/−^ tissue shown in green and unregulated genes shown in black. **B** Genes whose expression changed by ≥1.5-fold tissue in Plg-R_KT_^−/−^ tissue.

We used RNA Sequencing to investigate differences in gene expression profiles of Plg-R_KT_^−/−^ and Plg-R_KT_^+/+^ wound tissue harvested on day 11. Volcano plots are shown in Fig. 6a and genes whose expression changed by ≥ 1.5-fold with *p* < 0.05 are presented in Fig. 6b. Six of the genes that were downregulated are expressed in the epidermis (*HBA*½, *Csfr3*, *MMP3*, *Tnc*, and *Timp1*). Of downregulated genes, six were inflammation-related and eight were ECM related.

Of downregulated inflammation-related genes, six are expressed in myeloid cells (*Csf3r*, *Ilrl1*, *Cxcl5*, and *Ccr2*) and/or regulate macrophage function (*Il33*, *Cxcl5*, *Saa3*) and one is expressed in the epidermis (*Csfr3*). Of note, IL33 (and its receptor Il1rl1) accelerates the development of M2 macrophages in wound sites in vivo.

Of downregulated ECM-related genes, six are expressed in myeloid cells (*Mmp3*, *Serpine 1*, *Timp 1*, *Tnc*, and *Vcan*) or regulate macrophage function (*Postn*) and three are expressed in epidermis (*MMP3*, *Tnc*, and *Timp1*). Thus, deletion of Plg-R_KT_ has effects on the expression of genes involved in wound healing that are expressed by both myeloid cells and keratinocytes.

### Effect of fibrinogen depletion on wound healing in Plg-R_KT_^−/−^ mice

**Fig. 7:**
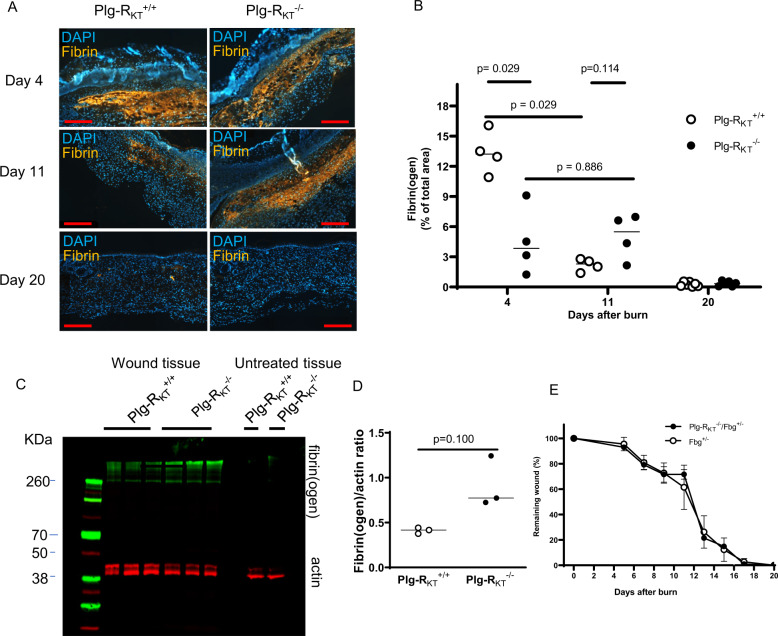
Enhanced fibrin(ogen) content in Plg-R_KT_^−/−^ wound tissue and the effect of fibrinogen heterozygosity on burn wound healing. **A** Immunohistochemical staining for fibrin in the tissue of Plg-R_KT_^−/−^ and Plg-R_KT_^+/+^ mice collected at different time points after induction of burn wounds (*n* = 4). Scale bar = 200 µm. Images were captured with a Zeiss Axio Imager Z1 (Zeiss, Oberkochen, Germany) or Nikon A1R Eclipse Ti-E inverted microscope (Nikon Instruments, Amsterdam, Netherlands). **B** Quantification of fibrinogen area in panel (**A**) (%) based on immunostaining using Image J software. Data analysis by robust ANOVA showed a significant effect for time (*p* < 0.0001, *f* = 68.694), a significant effect for genotype (*p* = 0.0002, *f* = 20.257) and a significant genotype × time interaction (*p* < 0.0001, *f* = 35.604). Post hoc testing by Mann–Whitney test: Day 4, Plg-R_KT_^+/+^ (Mdn = 13), Plg-R_KT_^−/−^ (Mdn = 3.8), (*U* = 0, *p* = 0.029), Hodges–Lehmann median difference = −9.3; Day 11, Plg-R_KT_^+/+^ (Mdn = 2.3), Plg-R_KT_^−/−^ (Mdn = 5.5), (*U* = 2, *p* = 0.114); Day 20, Plg-R_KT_^+/+^ (Mdn = 0.22), Plg-R_KT_^−/−^ (Mdn = 0.34), (*U* = 18, *p* = 0.731); Plg-R_KT_^+/+^ Day 4 (Mdn = 13.23), Day 11 (Mdn = 2.29), (*U* = 0, *p* = 0.029), Hodges–Lehmann median difference = -10.94; Plg-R_KT_^−/−^ Day 4 (Mdn = 3.842), Day 11 (Mdn = 5.500), *U* = 7, *p* = 0.886). Plg-R_KT_^+/+^ Day 4 (Mdn = 13.23), Day 20 (Mdn = 0.221), (*U* = 0, *p* = 0.0061), Hodges Lehmann median difference = −12.94); Plg-R_KT_^−/−^ Day 4 (Mdn = 3.842), Day 20 (Mdn = 0.344), (*U* = 0, *p* = .0095), Hodges–Lehmann median difference = −3.482. Medians and *p* values from post hoc testing by Mann–Whitney test is displayed. **C** Wound tissue obtained at day 11 after wounding or control abdominal tissue was lysed and 30 μg of total protein was electrophoresed under nonreducing conditions and western blotted with anti-fibrin(ogen) and anti-βactin antibodies. **D** Gels were scanned and quantified densitometrically using LICOR software. By Mann–Whitney test: Plg-R_KT_^+/+^ (Mdn = 0.42), Plg-R_KT_^−/−^ (Mdn = 0.77), (*U* = 0, *p* = 0.100), *n* = 3. Medians and *p* values determined by the Mann–Whitney test are displayed. **E** Quantification of the remaining wound area at different time points after burn-wounding of male Plg-R_KT_^−/−^/Fgn^+/−^ (*n* = 4) mice and Plg-R_KT_^+/+^/Fgn^+/−^ mice (*n* = 5). The mixed-effects model (REML) showed a significant effect of time (*p* = 0.001, *f* = 12.94), but no effect of genotype (*p* = 0.868, *f* = 0.030) and no genotype × time interaction (*p* = 0.762), *f* = 0.463).

Fibrin formation is an early hemostatic event following wounding that is then followed by subsequent plasmin-dependent fibrinolysis^31^. Plasminogen deficient mice exhibit impaired wound healing^12,14,18^ and genetic deletion of fibrinogen corrects the defect in skin wound healing in these mice^17^. We compared fibrin deposition during wound healing in Plg-R_KT_^−/−^ and Plg-R_KT_^+/+^ mice using immunohistochemistry. There was a significant difference between the genotypes in response to burn wounding with respect to time. Initially, at day 4, fibrin deposition in Plg-R_KT_^+/+^ wound tissue was significantly greater than in wound tissue of Plg-R_KT_^−/−^ littermates, consistent with relatively greater vascular permeability in Plg-R_KT_^+/+^ wound tissue^32^. However fibrin clearance from day 4 to day 11 was markedly and significantly impaired in Plg-R_KT_^−/−^ wound tissue compared with Plg-R_KT_^+/+^ wound tissue and, in addition, at day 11 there was a trend for an increased presence of fibrin in Plg-R_KT_^−/−^ compared with Plg-R_KT_^+/+^ wound tissue. Fibrin clearance was essentially complete in wounds of both genotypes at day 20 (Fig. 7a, b). Thus, the time course of fibrin clearance was impaired in Plg-R_KT_^−/−^ wound tissue. Western blot analysis also demonstrated increased fibrin content in Plg-R_KT_^−/−^ the tissue on day 11 (Fig. 7c, d). Remarkably, genetic reduction of fibrinogen levels of Plg-R_KT_^−/−^ mice to 50% completely abrogated the effect of Plg-R_KT_ deletion on the healing of burn wounds (Fig. 7e).

### Effects of concomitant deletion of Plg-R_KT_ and fibrinogen on gene expression during wound healing

**Fig. 8:**
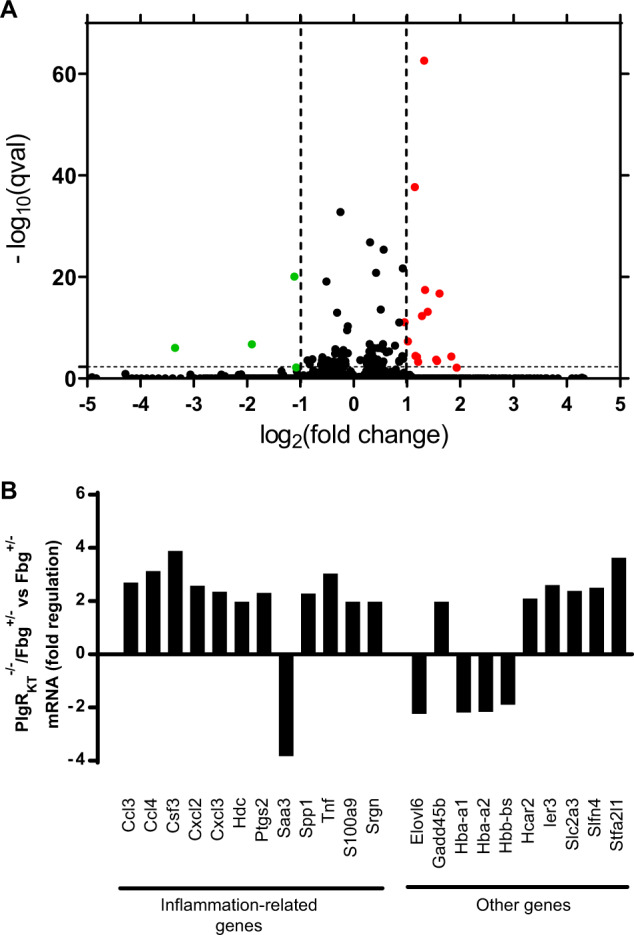
Gene expression in Plg-R_KT_^−/−^/fibrinogen^+/-^ and Plg-R_KT_^+/+^/fibrinogen^+/−^ wound tissue. mRNA sequencing of Plg-R_KT_^−/−^ and Plg-R_KT_^+/+^ wound tissue on a Fibrinogen^+/−^ (Fbg ^+/−^) genetic background was carried out on samples taken at day 11 (*n* = 4). All the genes had corrected *p* value ≤ 0.05. **A** Volcano plots with genes upregulated > 1.0-fold in Plg-R_KT_^−/−^/fibrinogen^+/−^ tissue shown in red, genes downregulated < 1.0 fold in Plg-R_KT_^−/−^/fibrinogen^+/−^ tissue shown in green and unregulated genes shown in black. **B** Genes whose expression changed by ≥1.5-fold tissue in Plg-R_KT_^−/−^/fibrinogen^+/−^ tissue.

We used mRNA sequencing to examine the effect of Plg-R_KT_ deletion on gene expression in the context of fibrinogen heterozygosity in wound tissue harvested at day 11. Volcano plots are shown in Fig. 8a and genes whose expression changed by ≥1.5-fold with *p* < 0.05 are presented in Fig. 8b. Strikingly, eleven inflammation-related genes were up-regulated whose expression had not been affected in Plg-R_KT_^−/−^ wound tissue with wild-type levels of fibrinogen. The fibrinogen^+/−^ background did not influence the effect of Plg-R_KT_ deletion on expression of Saa3 or of hemoglobin. Therefore, the presence of fibrin(ogen) appears to be necessary for Plg-R_KT_ to exert its ability to promote wound healing.

## Discussion

The present study provides mechanistic insight into the novel role of the plasminogen receptor, Plg-R_KT_, in cutaneous wound healing. Plg-R_KT_ exerted pleiotropic, interrelated effects in the inflammatory, proliferative, and remodeling phases of wound healing. We found that (1) Plg-R_KT_ was expressed in epidermis and expression increased during wound healing; (2) deletion of Plg-R_KT_ decreased the rate of healing of burn wounds; (3) Plg-R_KT_ regulated plasminogen accumulation in burn wounds; (4) Plg-R_KT_ regulated expression of inflammatory cytokines during wound healing; (5) specific deletion of Plg-R_KT_ in myeloid cells impaired wound healing during the transition from the inflammatory to proliferation phase; (6) specific deletion of Plg-R_KT_ in keratinocytes accelerated wound closure; (7) Plg-R_KT_ regulated fibrin deposition in wounds; and (8) fibrin(ogen) levels modulated regulation of gene expression by Plg-R_KT._

Plg-R_KT_ was initially discovered in monocytes/macrophages^19^. Here, we found expression, also, in isolated primary keratinocytes as well as in keratinocytes and hair follicles within epidermal tissue. Notably, expression of Plg-R_KT_ in keratinocytes increased as wound healing progressed, consistent with a role for Plg-R_KT_ in the wound-healing program. This is the first example of changes in tissue Plg-R_KT_ expression during the progression of a pathophysiological program.

Healing of cutaneous wounds is impaired in plasminogen deficient mice^12,15,17^. We have demonstrated previously that plasminogen bound to macrophages and neutrophils, is transported to the wound area, where the level of plasminogen is increased locally. This leads to the induction of intracellular signaling and cytokine release^18^. Thus, the role of plasminogen in the initial stages of inflammation is predominantly induction of intracellular signaling^18^. In the current study, deletion of the plasminogen receptor, Plg-R_KT_, significantly attenuated the rate of wound healing. And, when either plasminogen deficient or Plg-R_KT_^−/−^ mice were treated with anti-Plg-R_KT_ mAB, followed by exogenous plasminogen, the accumulation of plasminogen in wounds was decreased. Furthermore, in Plg-R_KT_^−/−^ wound tissue, the expression of six inflammation-related genes that are expressed by myeloid cells (*Csf3r*, *Ilrl1*, *Cxcl5*, and *Ccr2*) and/or regulate macrophage function (*Il33*, *Cxcl5*, *Saa3*) was downregulated. These results are consistent with the concept that Plg-R_KT_ is a key regulator of the effect of plasminogen on immune cell function in the healing wound. Of note, IL33 (and its receptor Il1rl1) accelerates the development of M2 macrophages in wound sites in vivo^33^. We have shown previously that plasminogen and Plg-R_KT_ promote polarization of macrophages to an M2-like phenotype^25,34^. Thus, in addition to dysregulated cytokine expression, decreased development of M2-like macrophages may also contribute to the delayed healing curve in Plg-R_KT_^−/−^ mice, resulting in a longer pro-inflammatory phase. Earlier studies in vitro documented plasmin-dependent stimulation of intracellular signaling pathways and cytokine release by monocytes and macrophages that depend on the interaction of plasmin with cell surfaces^35–37^ and our recent studies suggest that Plg-R_KT_ can mediate plasmin(ogen)-dependent intracellular signaling and cytokine release^24,25^. The current study shows that Plg-R_KT_ regulates the expression of many cytokines that were not previously known to be regulated by Plg-R_KT_. It is noteworthy that Plg-R_KT_ contains only a four amino acid cytoplasmic domain. Thus, signaling mediated by Plg-R_KT_ is likely to involve an interaction with additional transmembrane molecules, similar to the example of the GPI-linked urokinase receptor (uPAR) that is able to mediate intracellular signaling^38^.

Despite functioning to transport plasminogen to the wound to promote cytokine release, recruitment of immune cells to cutaneous wounds is not affected by plasminogen deficiency^15,18^ and we did not find an effect of Plg-R_KT_ deficiency on recruitment of macrophages and neutrophils to wounds. Nonetheless, we did find a significant decrease in the rate of wound healing when Plg-R_KT_ was specifically deleted in myeloid cells, consistent with a role for Plg-R_KT_-bound plasmin(ogen) in regulating cytokine release as well as macrophage polarization. The lack of effect of Plg-R_KT_ and plasminogen on recruitment of macrophages to wound sites is notable considering the requirement for both Plg-R_KT_ and plasminogen in experimental peritonitis^21,23,39^. Conversely, increased macrophage infiltration is observed in spontaneously thrombotic organs of plasminogen deficient mice^30,40^ and in mammary glands of Plg-R_KT_^−/−^ mice^26^. Thus, the roles of both plasminogen and Plg-R_KT_ in macrophage recruitment depend on the pathophysiological setting.

Plasmin directly promotes keratinocyte migration in vitro^16^ and keratinocyte migration is decreased in cutaneous wounds in plasminogen deficient mice^12,17^. In Plg-R_KT_^−/−^ mice the area of the keratinocyte tongue protruding at wound edges was decreased at day 4, consistent with altered keratinocyte migration. Decreased keratinocyte migration may result from impaired ability to degrade the ECM in order to migrate due to decreased plasmin associated with the cell surface in the absence of Plg-R_KT_, as well as a dysregulated ECM due to impaired fibrin clearance and resulting from down-regulation of other ECM-related genes in Plg-R_KT_^−/−^ wound tissue (*Mmp3*, *Serpine 1*, *Timp 1*, *Tnc*, and *Vcan*).

Following wound closure (day 20), the epidermal thickness was significantly less in Plg-R_KT_^−/−^ tissue. There also was a trend for decreased epidermal thickness in kPlg-R_KT_^−/−^ mice. Paradoxically, deletion of Plg-R_KT_ specifically in keratinocytes significantly accelerated the rate of healing (determined by wound closure) during the proliferation phase. Mechanistically, only two genes were upregulated in Plg-R_KT_^−/−^ compared with Plg-R_KT_^+/+^ wound tissue, filaggrin (*Flg*), and caspase 14 (*Casp14*). Filaggrin is essential for the regulation of epidermal homeostasis and is a marker for keratinocyte differentiation, thus higher filaggrin expression is correlated with lower proliferation^41^. Caspase 14 is a non-apoptotic caspase involved in epidermal differentiation and is the predominant caspase in epidermal stratum corneum^42^. Caspase 14 plays a role in keratinocyte differentiation and is required for cornification and regulates maturation of the epidermis by proteolytically processing filaggrin. Thus, in Plg-R_KT_^−/−^ wounds, the thinner epidermis at day 20 and earlier wound closure may be attributed to, at least in part, to increased differentiation and decreased proliferation of keratinocytes, consistent with increased filaggrin and caspase 14 expressions.

Plasmin is the major enzyme responsible for fibrinolysis^30,40^. Initially (day 4) fibrin deposition was significantly greater in wound tissue of Plg-R_KT_^+/+^ littermates. The early increase in fibrin deposition in Plg-R_KT_^+/+^ mice may be due to relatively greater vascular permeability in Plg-R_KT_^+/+^ wound tissue^32^. Subsequently, clearance of the initial fibrin deposits in Plg-R_KT_^+/+^ wound tissue took place, while fibrin clearance was delayed in Plg-R_KT_^−/−^ wounds. Genetic reduction of fibrinogen levels of Plg-R_KT_^−/−^ mice to 50% completely reversed the effect of Plg-R_KT_ deletion on the healing of burn wounds. This parallels the rescue of defective cutaneous wound healing in plasminogen deficient mice by concomitant deletion of fibrinogen^17^. Remarkably, the effect of Plg-R_KT_ deletion on gene expression in wound tissue was markedly altered on the fibrinogen heterozygous background. Strikingly, 11 inflammation-related genes were upregulated. Extravascular fibrin(ogen) is a strong modifier of proinflammatory disease, acting through the integrin α_M_β_2_ on macrophages^43–46^. The upregulation of *Ccl3*, *Ccl4*, *Cxl2*, *Cxc13*, and *Tnf* may result from decreasing an effect of persistent fibrin(ogen) on macrophages and is potentially a key factor in the ability of fibrin(ogen) deletion to reverse the defective wound healing phenotype of Plg-R_KT_^−/−^ mice. Thus, defective fibrinolysis is likely to play a role in reduced keratinocyte migration as well as impaired stimulation of cytokine release by macrophages.

In summary, our results suggest that Plg-R_KT_ has multiple functions in the wound-healing program (as outlined in Supplementary Table 1). Plg-R_KT_ promotes plasminogen transport to the wound site and promotes plasmin(ogen)-dependent cytokine release as a key step in the proinflammatory phase of wound healing. Plg-R_KT_ regulates the composition of the ECM, regulates gene expression by keratinocytes, and promotes proliferation of keratinocytes while decreasing keratinocyte differentiation, as key steps in the proliferation and resolution phases. Fibrinolysis, regulated by Plg-R_KT_, is a necessary (although not sufficient) step in the wound healing program that impacts both cytokine release by macrophages, keratinocyte migration, and ECM remodeling. The results of our study may also apply more broadly to healing in other tissue due to other types of injury including infection and inflammation.

## Supplementary information

Supplementary Figure S1

Supplementary Table 1

Supplementary Methods
