# Supplementary figures and images for: The plasminogen receptor, Plg-RKT, plays a role in inflammation and fibrinolysis during cutaneous wound healing in mice
Source: Cell Death Dis. 2020 Dec 12;11(12):1054. doi: 10.1038/s41419-020-03230-1 (PMC7733490; doi:10.1038/s41419-020-03230-1)

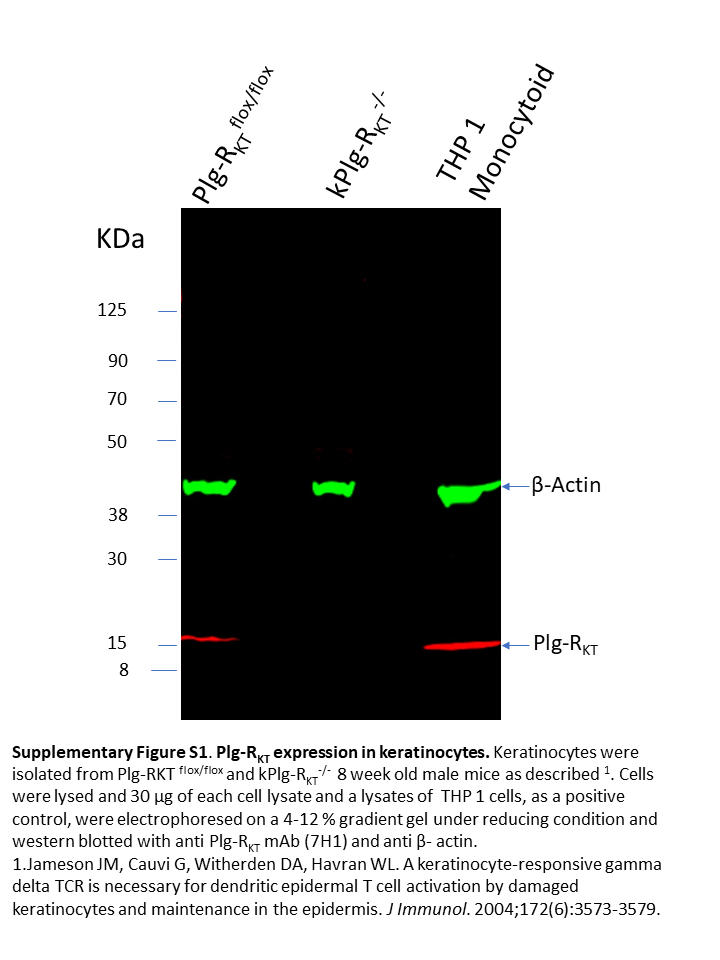

Supplement: Supplementary file 1 — Supplementary Figure S1 [file 41419_2020_3230_MOESM1_ESM.tif]

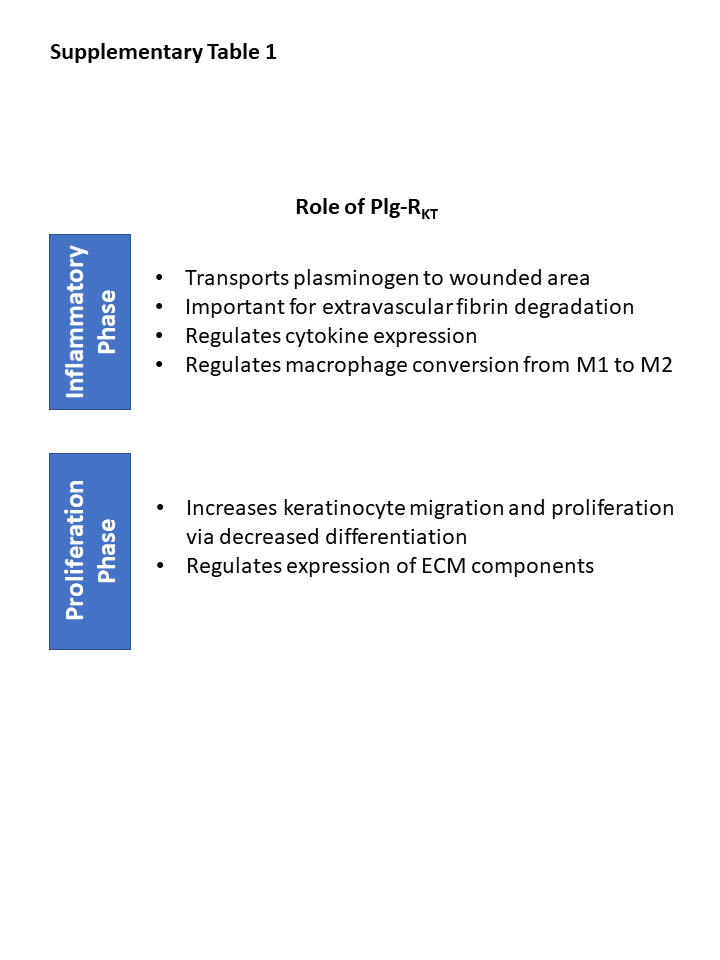

Supplement: Supplementary file 2 — Supplementary Table 1 [file 41419_2020_3230_MOESM2_ESM.tif]
