# Supplementary material for: The plasminogen receptor, Plg-RKT, plays a role in inflammation and fibrinolysis during cutaneous wound healing in mice
Source: Cell Death Dis. 2020 Dec 12;11(12):1054. doi: 10.1038/s41419-020-03230-1 (PMC7733490; doi:10.1038/s41419-020-03230-1)
Supplement: Supplementary file 3 — Supplementary Methods [file 41419_2020_3230_MOESM3_ESM.docx]

**Supplementary Methods**

**Proteins and ELISA Kits**

Plasminogen was purchased from Omnio AB (human Glu-plasminogen in 137 mM NaCl, 2.7 mM KCl, 10 mM Na_2_HPO_4_, 1.8 mM KH_2_PO_4_ defined as PBS buffer, Omnio AB, Umeå, Sweden) and was labeled with Alexa 488 (Invitrogen AB, Lidingö, Sweden) according to the manufacturer’s instruction. The labeling efficiency was 40%. The pan-specific mouse anti-Plg-R_KT_ mAb (7H1) was developed in our laboratory.^1^ Rat IgG2a , MCA1212 was from Biorad (Oxford, UK), the mouse plasminogen ELISA kit was from Omnio, Umeå, Sweden, the mouse IgG2a isotype control, the mouse IL-6 ELISA and TNFα ELISA kits were from Invitrogen (Carlsbad, CA,USA).

**Immunohistochemistry and Histology**

For immunohistochemistry, slides were deparaffinized in xylene, rehydrated in graded ethanol washes, incubated in citrate buffer at 95°C for antigen retrieval, and blocked with serum-free protein block *(*DAKO*,* Carpinteria, CA, USA) and Avidin (Vector Laboratories, Burlingame, CA*).* First and second antibodies were goat anti-mouse fibrin(ogen) antibody (REF.No [GAM/Fbg](https://www.nordicmubio.com/product/goat-anti-mouse-fibrinogen/), Nordic Immunological Lab, Tilburg, Netherlands) followed by rabbit anti-goat antibodies conjugated with Alexa Fluor 555 (REF.No A21431, Thermo Fisher Scientific, Waltham, MA, USA); rat anti-mouse Ly-6B.2 monoclonal antibody clone 7/4 (REF.No MCA771G, AbD Serotec, Oxford, UK) followed by biotinylated donkey anti-rat IgG antibodies (REF.No **AS10 1093** Agrisera, Vännäs, Sweden) and streptavidin conjugated with Alexa Fluor 647 (REF.No S32357, Thermo Fisher Scientific, Waltham, USA); rabbit anti-mouse CD68 (ab125212, Abcam, Cambridge UK) followed by goat anti-rabbit IgG (H+L) conjugated with Dylight 594 *(*REF.No DI-1594 *,* Vector Laboratories, Burlingame, CA). Mouse anti-Plg-R_KT_ mAb (7H1) was directly labelled with Alexa Fluor 555 using an antibody labelling kit (Thermo Fisher Scientific). Nuclei were stained with DAPI (Thermo Fisher Scientific). For histology, 3 μm sections were stained with hematoxylin & eosin. Images were captured with a Zeiss Axio Imager Z1 (Zeiss, Oberkochen, Germany) or Nikon A1R Eclipse Ti-E inverted microscope (Nikon Instruments, Amsterdam, Netherlands). Quantification of fluorescent areas was performed using Image J software.

For histological analyses, the sections were stained with Mayer’s hematoxylin (Histolab, Gothenburg, Sweden) and images were taken using a Leica DC300F digital camera attached to a Leica DM LB microscope (Leica, Wetzlar, Germany). Epidermal thickness was measured from the photos using Adobe Photoshop.

**Western blotting**

Wound tissue was lysed in RIPA buffer with anti-protease and anti-phosphatase cocktail (Thermo Fisher Scientific) and electrophoresed on 4-15% polyacrylamide-SDS gels under non-reducing conditions. Following electrotransfer, nitrocellulose membranes were incubated with anti-human fibrin(ogen) (Abcam ab92572, Cambridge, MA) and the loading control, anti-βactin (Li-COR P/N 926-42212, Licoln, NE), washed with PBS-containing 0.1% Tween-20 and incubated with species specific IRDye^®^680RD/800CW-conjugated secondary antibodies. Immunoreactive bands were visualized using the Odyssey Imaging System (LI-COR), according to the manufacturer’s instructions (Biosciences). For densitometry, membranes were scanned and quantified using Image Studio™ Lite Software 5.2 (LI-COR).

**Quantitative RT-PCR**

Skin samples from the wounded area and control unwounded skin (100–200 mg) were cut into 1-2 mm^2^ pieces and kept in RNAlater (Thermo Fischer Scientific) for 3 days at +4°C. The samples were homogenized in TRIzol (Ambion, Carlsbad CA, USA) in Precellys CK28R tubes on a Precellys 24 homogenizer (from Bertin Technologies, Lyon, France). Total RNA was extracted using the PureLink RNA Mini Kit (Ambion). Aliquots of 2.5 µg total RNA were reverse-transcribed using a SuperScript VILO cDNA Synthesis Kit (Invitrogen) and diluted 3-fold with diethylpyrocarbonate-treated H_2_O. Quantitative real-time PCR was performed using the comparative C_T_ method and with TATA-binding protein mRNA as the internal reference gene. Gene-specific primers and probes (TaqMan Gene Expression Assays) were from Applied Biosystems (Foster City, USA), and 2× SsoAdvanced Universal Probes Supermix was from Bio-Rad (CA, USA). Samples were run in triplicate on a StepOnePlus Instrument (Applied Biosystems) using the real-time PCR conditions recommended by the manufacturers of the 2× SsoAdvanced Universal Probes Supermix.

**mRNA Sequencing**

For mRNA sequencing, skin samples were incubated in RNAlater and homogenized in QIAzol (Qiagen, Hilden, Germany) using Precellys CK28R tubes on a Precellys 24 homogenizer. Total RNA was extracted with the RNeasy Lipid Tissue Mini Kit (Qiagen) according to the manufacturer’s instructions. The purity of the RNA from each individual mouse was checked with a NanoDrop 2000 spectrophotometer (Thermo Scientific), and the OD260/280 and OD260/230 were ≥ 2.0 for all the samples. The RNA quality was analyzed using a High Sensitivity RNA Analysis Kit, 15nt (Advanced Analytical, Santa Clara, USA) together with the Fragment Analyzer. RNA quality values were ≥ 6.8 for all samples. RNA samples for each treatment group and time point were pooled, and 5 µg of the pooled RNA was sent to Novogene (Hong Kong) for transcriptome sequencing and data analysis. A gene was assumed to be differentially expressed if there was at least a two-fold difference in the expression between different genotypes and corrected p-value was ≤0.05.

1. Lighvani S, Baik N, Diggs JE, Khaldoyanidi S, Parmer RJ, Miles LA. Regulation of macrophage migration by a novel plasminogen receptor Plg-RKT. *Blood*. 2011;118(20):5622-5630.
